# Ten-eleven translocation 2-mediated DNA demethylation plays a positive regulatory role in BMP9-induced osteogenic differentiation of mesenchymal stem cells

**DOI:** 10.1016/j.gendis.2026.102116

**Published:** 2026-02-28

**Authors:** Connie Chen, Yi Zhu, Sarina Zhao, Michelle Xiang, Chao Yu, Yan Peng, Yuting Liang, Jeffrey Baek, Longwei Qiao, Xiangyu Dong, Fangyi Yao, Jinglin Huang, Tong-Chuan He, Russell R. Reid, Jiaming Fan, Gregory Schimizzi, Sofia Bougioukli, Yong Xu, Changqi Luo

**Affiliations:** a Molecular Oncology Laboratory, Department of Orthopaedic Surgery and Rehabilitation Medicine, The University of Chicago Medical Center, Chicago, IL 60637, USA; b Department of Orthopaedic Surgery, The First Affiliated Hospital of Soochow University, Orthopaedic Institute, Soochow University, Suzhou, Jiangsu 215006, China; c Departments of Orthopaedic Surgery, Cardiology, and Obstetrics and Gynecology, The Affiliated University-town Hospital, Chongqing Medical University, Chongqing 401331, China; d Center for Clinical Laboratory, The First Affiliated Hospital of Soochow University, Suzhou, Jiangsu 215006, China; e Center for Reproduction and Genetics, The Affiliated Suzhou Hospital of Nanjing Medical University, Suzhou, Jiangsu 215000, China; f Ministry of Education Key Laboratory of Diagnostic Medicine, and Department of Clinical Biochemistry, School of Laboratory Diagnostic Medicine, Chongqing Medical University, Chongqing 400016, China; g Department of Clinical Laboratory Medicine, The Second Affiliated Hospital of Nanchang University, Nanchang, Jiangxi 330006, China; h Laboratory of Craniofacial Biology and Development, Section of Plastic and Reconstructive Surgery, Department of Surgery, The University of Chicago Medical Center, Chicago, IL 60637, USA; i Department of Orthopaedic Surgery, The Second People's Hospital of Yibin, Affiliated with West China School of Medicine, Yibin, Sichuan 644000, China

Mesenchymal stem cells (MSCs) respond to diverse growth factors and signaling cascades that coordinately regulate their osteogenic differentiation potential. Bone Morphogenetic Protein 9 (BMP9), a member of the TGF-β superfamily, is one of the most potent inducers of osteogenic differentiation of MSCs.^1^ DNA methylation plays a role in osteogenesis as an epigenetic regulatory mechanism. Previous studies demonstrated that hypermethylation of the BMP2 promoter regions silenced osteogenic genes.^2^ The tet-eleven translocation (Tet) family of dioxygenases promotes DNA demethylation through oxidizing 5-methylcytosine (5-mC) to 5-hydroxymethylcytosine (5-hmC) and its derivatives in an Fe(II)/α-ketoglutarate-dependent oxidation reaction.^3^ While all three TET family members (Tet1, Tet2, Tet3) are expressed in MSCs, Tet2 has been identified as the most significantly upregulated member upon osteogenic induction and is critically involved in lineage determination. Furthermore, studies have shown that Tet2, rather than Tet1 or Tet3, plays a dominant role in the transcriptional activation of key osteogenic factors like *Runx2*.^4^ Here, by employing a multiplex-siRNA system to effectively silence Tet2 expression in MSCs, we demonstrate that the loss of Tet2 expression significantly hampers BMP9-induced osteogenic differentiation and ectopic bone formation, confirming our hypothesis in which Tet2-mediated DNA demethylation plays a significant regulatory role in osteogenic differentiation of MSCs.

Although BMP9 is a well-established inducer of osteogenesis, epigenetic regulation of its osteogenic signaling remains unclear. Given Tet2's role in promoting DNA demethylation and regulating osteogenesis, we postulated that Tet2 may serve as a positive regulator of BMP9-induced osteogenic differentiation of MSCs. Here, a multiplex-siRNA silencing system was constructed as an adenovirus-mediated Tet2 knockdown (KD) vector (Ad-SimTet2) (Table S1). Osteogenic changes following Tet2 KD were analyzed both *in vitro* and *in vivo* by infecting immortalized mouse bone marrow-derived MSCs (imBMSCs) with Ad-GFP plus Ad-RFP (negative control), Ad-SimTet2 plus Ad-GFP, Ad-BMP9 plus Ad-RFP, and a co-infection of Ad-SimTet2 and Ad-BMP9.

To confirm the KD efficacy of Ad-SimTet2, we infected imBMSCs with Ad-SimTet2 and performed touchdown quantitative real-time PCR (TqPCR) (Table S2). Compared to the Ad-GFP control, Tet2 mRNA levels were significantly reduced in the Ad-SimTet2 group (Fig. 1A), confirming the silencing efficiency of Ad-SimTet2. Alkaline phosphatase (ALP) staining and activity were assessed as an early marker of osteogenic differentiation (Fig. 1B). ALP staining results revealed that by Day 3, cells treated with BMP9 exhibited apparent ALP-positive signals, whereas those in the BMP9 and Ad-SimTet2 group showed noticeably weaker staining. The Ad-SimTet2-alone group exhibited minimal ALP activity, comparable to that of the GFP control. A similar staining pattern was observed at Day 7. Quantitatively, ALP activity was assessed using a bioluminescence assay. The results showed that co-infection with Ad-SimTet2 led to a significant reduction of ALP activities, compared to that with BMP9 alone, suggesting that Tet2 knockdown may inhibit BMP9-induced osteogenic signaling.

**Figure 1:**
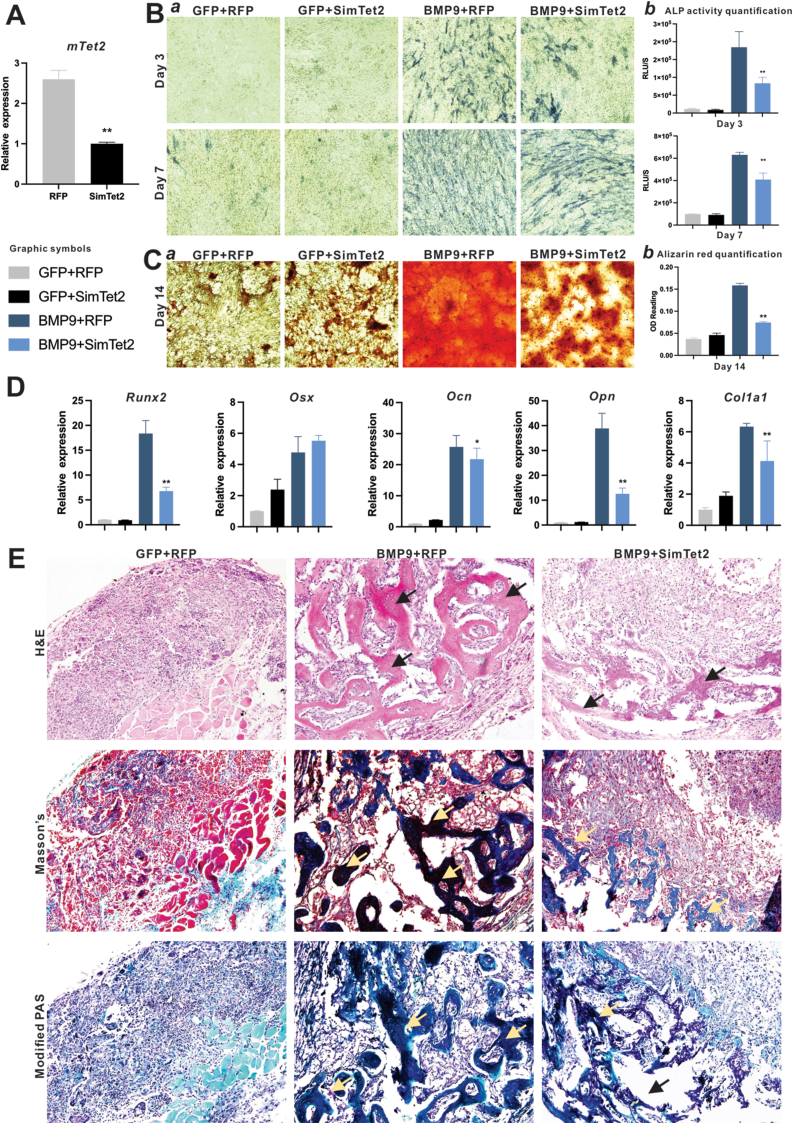
Tet2 knockdown (KD) reduces BMP9-induced osteogenic differentiation. **(A)** Verification of Tet2 KD in immortalized mouse bone marrow-derived mesenchymal stem cells (imBMSCs) by touchdown quantitative real-time PCR (TqPCR). Multiplex siRNA vector Ad-SimTet2 decreases expression of Tet2 in imBMSCs. TqPCR reactions were performed in triplicate. ∗∗*P* < 0.01, compared with the negative control group. **(B)** Early osteogenic marker alkaline phosphatase (ALP) activity. Subconfluent imBMSCs were infected with Ad-GFP plus Ad-RFP (negative control), Ad-Ad-SimTet2 plus Ad-GFP, Ad-BMP9 plus Ad-RFP, and a coinfection of Ad-SimTet2 and Ad-BMP9. Qualitative histochemical analysis **(*a*)** or quantitative measurement **(*b*)** of ALP activity was determined at days 3 and 7. Representative results are shown. ∗∗*P* < 0.01 compared with the BMP9 group. **(C)** Mineralized matrix formation. Subconfluent imBMSCs were transduced with the same group setting as described in Figure 1B, and cultured in mineralization medium for 14 days, followed by alizarin red S staining **(*a*)**. The stained mineral nodules were dissolved and quantified **(b)**. ∗∗*P* < 0.01, compared with the BMP9 group. **(D)** Expression of osteogenic markers *Runx2*, *Osx*, *Ocn*, *Opn,* and *Col1a1* in imBMSCs. Subconfluent imBMSCs were transduced with same group setting as described in Figure 1B and collected at 48 h after infection. Total RNA was isolated from the infected cells and subjected to RT-PCR reaction, followed by TqPCR analysis of osteogenic regulators *Runx2* and *Osx*, and late osteogenic markers of *Ocn*, *Opn*, and *Col1a1*. TqPCR reactions were performed in triplicate. ∗*P<* 0.05, ∗∗*P* < 0.01, compared with the BMP9 group. **(E)** Effect of Tet2 KD effect on BMP9-induced bone formation *in vivo*. Subconfluent imBMSCs were transduced with same group setting as described in Figure 1B and collected at 48 h after infection, followed by subcutaneously injected into the flanks of athymic nude mice. Masses were retrieved at 4 weeks post implantation, decalcified and paraffin-embedded for Hematoxylin and Eosin, Masson's trichome, or modified periodic acid-Schiff staining. Arrows indicate mature trabecular bone formation.

Mineralization assays were performed to further examine the impact of Tet2 KD on the late stage of osteogenic differentiation upon BMP9 stimulation. Alizarin Red staining of calcium mineral deposits was recorded under bright-field microscopy (Fig. 1C, panel *a*). We found that Alizarin Red staining was inhibited by Tet2 KD, with the coinfection group showing over 50% less calcium deposition than the Ad-BMP9-alone group (*P* < 0.01) (Fig. 1C, panel *b*). Expression of five osteogenic markers was assessed using TqPCR (Fig. 1D). Tet2 KD deregulated the expression of osteogenic markers. Specifically, the expression of osteogenic markers *Runx2*, *Ocn*, *Opn*, and *Col1a1* was lower in the Ad-SimTet2 coinfection group than in the Ad-BMP9 positive control group. Collectively, these results indicate that Tet2 KD impairs the osteogenic differentiation of imBMSCs, suggesting that functional levels of Tet2 may benefit BMP9-induced osteogenesis.

To further characterize the *in vivo* effects of Tet2 knockdown, imBMSCs transduced with different combinations of adenoviruses were resuspended in a previously characterized biodegradable thermoresponsive scaffold poly (polyethylene glycol citrate-co-N-isopropylacrylamide) gel (PPCNg), and subcutaneously injected into the flanks of athymic nude mice. At 28 days post injection, the mice were euthanized, and the resultant masses were retrieved. The relative volumes of these masses were consistent with the trends observed in ALP activity, with the Ad-BMP9 group being the largest, followed by the co-infected group, while the Ad-GFP group and the Ad-SimTet2 group being the smallest.

The retrieved masses were fixed, paraffin-embedded, and subjected to Hematoxylin and Eosin (H&E) staining, Masson's trichrome staining, and modified periodic acid-Schiff (PAS) staining (Fig. 1E). Histological analysis showed masses from the Ad-GFP negative control group showed mostly fibrous tissue, residual PPCNg scaffold materials, and undifferentiated MSCs, with no obvious osseous tissue (Fig. 1E, panel *a*). In the Ad-BMP9 group, H&E staining showed dramatic tubercle bone formation (indicated by black arrows and dark pink areas). Masson's trichrome staining revealed mature bone formation (yellow arrows and dark red areas), whereas modified PAS staining showed bone formation (yellow areas and green areas) (Fig. 1E, panel *b*). In contrast, in the Ad-BMP9 and Ad-SimTet2 group, H&E staining revealed significantly smaller areas of tubercle bone formation, and Masson's trichrome and modified PAS staining showed immature bone compared to the Ad-BMP9 group (Fig. 1E, panel *c*). Overall, the Ad-BMP9 and Ad-SimTet2 coinfection exhibited a much smaller quantity of bone formation and less mature bone matrix formation than the Ad-BMP9 group, indicating Tet2 KD hampered BMP9-induced osteogenic differentiation. Collectively, our *in vitro* and *in vivo* findings demonstrate that Tet2 KD impaired BMP9-induced osteogenesis, suggesting Tet2 plays a potential positive regulatory role in BMP9-intiated osteogenic signaling in MSCs. It is noteworthy that Tet2 KD in other MSCs, such as adipose-derived MSCs, yielded similar results.

BMP9 signals through type I receptors ALK1/ALK2 and type II receptors (BMPRII/ActRII) to activate Smad1/5/8, and–unlike other osteogenic BMPs (e.g. BMP2, BMP4, BMP6, and BMP7)–is uniquely resistant to inhibitors such as noggin and BMP3, resulting in prolonged osteoinductive signaling.^1^ BMP9 also signals through the non-canonical BMPR-Smad-independent pathway, involving various MAPKs, allowing BMP9 to induce angiogenesis, neurogenesis, osteogenesis, and adipogenesis.^1^ Its high potency also stems from its extensive crosstalk with Wnt/β-catenin, Notch, IGF-2, HIF-1α, and other growth factor signaling axes.^1^

Tet2 regulates lineage commitment across multiple systems, as evidenced by the increased repopulating capacity and impaired differentiation in Tet2-mutant hematopoietic stem cells, as well as increased Tet2 and 5-hmC levels during smooth muscle cell differentiation. Previous research has shown that Tet2 expression plays a role in the osteogenic differentiation of MSCs; however, the mechanism by which Tet2 regulates osteogenesis required further elucidation, and its regulatory role in BMP9-induced osteogenic differentiation of MSCs has not been studied. Recent work has shown that Tet2/Tet3 can act upstream to activate *bmp4* expression through a TET–Sall4 regulatory axis, suggesting that Tet activity may modulate BMP signaling rather than being downstream of it. It has been proposed that Tet1 and Tet2 regulate osteogenesis through the demethylation of the *P2rX7* promoter.^5^ Tet1 and Tet2 deficiency results in reduced demethylation of the *P2rX7* promoter, leading to downregulated exosome release. This impaired exosome secretion causes the accumulation of miRNAs that inhibit *Runx2* signaling, a critical pathway for osteogenic differentiation, and subsequently decreases osteogenic differentiation. Tet2 was also found to promote BMSC differentiation into the osteoblast fate partly through transcriptional activation of *Runx2*.^4^

In conclusion, Tet2 KD reduces BMP9-induced osteogenesis. Although the precise mechanism by which Tet2 interacts with the BMP9 signaling pathway remains to be fully elucidated, the known regulation of osteogenic genes via 5hmC levels and the regulation of miRNA expression by Tet2 collectively demonstrate its role in modulating the epigenetic landscape to influence osteogenesis. Further investigation is required to delineate the detailed mechanisms through which Tet2 regulates BMP9 osteogenic signaling in MSCs.

## CRediT authorship contribution statement

**Connie Chen:** Writing – original draft, Formal analysis, Data curation. **Yi Zhu:** Writing – original draft, Funding acquisition, Formal analysis, Data curation. **Sarina Zhao:** Writing – original draft, Data curation. **Michelle Xiang:** Writing – original draft, Formal analysis. **Chao Yu:** Methodology, Formal analysis. **Yan Peng:** Methodology, Formal analysis. **Yuting Liang:** Methodology. **Jeffrey Baek:** Methodology. **Longwei Qiao:** Writing – review & editing, Methodology. **Xiangyu Dong:** Writing – review & editing. **Fangyi Yao:** Writing – review & editing. **Jinglin Huang:** Methodology. **Tong-Chuan He:** Writing – review & editing, Project administration, Funding acquisition, Conceptualization. **Russell R. Reid:** Writing – review & editing, Supervision, Conceptualization. **Jiaming Fan:** Writing – review & editing, Conceptualization. **Gregory Schimizzi:** Writing – review & editing, Conceptualization. **Sofia Bougioukli:** Writing – review & editing, Supervision, Methodology, Formal analysis, Conceptualization. **Yong Xu:** Writing – review & editing, Project administration, Data curation, Conceptualization. **Changqi Luo:** Writing – review & editing, Funding acquisition, Conceptualization.

## Ethics declaration

The care and use of animals were approved by the Institutional Animal Care and Use Committee (IACUC) of The University of Chicago (No. ACUP #71105). All experimental procedures followed the approved guidelines.

## Data availability

The relevant data and its supplemental data can be found in the article or obtained from the corresponding author upon request.

## Funding

The reported study was supported in part by the Health Commission of Sichuan Province Medical Science and Technology Program (China) (No. 24QNMP047 to CQL). This project was also supported in part by core facilities grants from The University of Chicago Cancer Center Support Grant (No. P30CA014599) and the National Center for Advancing Translational Sciences of the National Institutes of Health through Grant Number UL1 TR000430. YZ was supported by Postdoctoral Fellowship Program of CPSF (China) (No. GZC20251571) and Jiangsu Funding Program for Excellent Postdoctoral Talent (China) (No. 2025ZB269). The funding sources were not involved in the study design; in the collection, analysis, and interpretation of data; in the writing of the report; and in the decision to submit the paper for publication.

## Conflict of interests

Tong-Chuan He is the member of Genes & Diseases Editorial Board. To minimize bias, he was excluded from all editorial decision-making related to the acceptance of this article for publication. The remaining authors declare no conflict of interests.
