# Supplementary material for: Ten-eleven translocation 2-mediated DNA demethylation plays a positive regulatory role in BMP9-induced osteogenic differentiation of mesenchymal stem cells
Source: Genes Dis. 2026 Feb 28;13(4):102116. doi: 10.1016/j.gendis.2026.102116 (PMC13015237; doi:10.1016/j.gendis.2026.102116)
Supplement: Multimedia component 1 [file mmc1.docx]

**SUPPLEMENTAL MATERIALS**

**Methods and Materials**

Chemicals and Cell Culture

Human HEK-293 cells were obtained from the American Type Culture Collection (ATCC,

Manassas, VA). 293pTP, RAPA and 293GP cells were derived from HEK-293 cells as previously described^1-3^. Immortalized mouse bone marrow mesenchymal stem cells (imBMSCs) were previously characterized^4^. All cells were cultured in the DMEM supplemented with 10% fetal bovine serum (FBS, Gemini Bio-Products), 100 U/mL penicillin, and 100 μg/mL streptomycin at 37 °C in 5% CO2 as described. Unless indicated otherwise, chemicals were obtained from Thermo Fisher Scientific (Waltham, MA) or Millipore Sigma (St. Louis, MO).

Construction and Amplification of Recombinant Adenoviral Vectors

Ad-SimTet2 was constructed using FAMSi system^5^. Briefly, siRNAs were PCR amplified (**Table S1**), preassembled, and cloned into the shuttle vector (pSiEB) and subsequently cloned into the adenoviral vector (pAddRD), followed by package and amplification using 293pTP and HEK-293 cells. Ad-GFP, Ad-RFP and Ad-BMP9 were constructed using AdEasy technology as previously described^6,7^. Polybrene (4-8 ug/mL) was used to enhance adenoviral infection efficiency.

RNA Isolation and Touchdown qPCR (TqPCR)

RNA was isolated from cultured cells using the NucleoZOL reagent (Takara Bio USA Inc.) and subjected to reverse transcription into cDNA with hexamer and M-MuLV reverse transcriptase (New England Biolabs, Ipswich, MA). cDNA was diluted and used as PCR primers, PCR primers were designed to amplify the genes of interest (**Table S2**), and RT products were diluted and used as templates in SYBR Green-based touchdown qPCR (TqPCR) analysis^8,9^. Reactions were run in triplicate. Gene expression was normalized with *β-Actin* expression level and calculated by using the 2^–ΔΔCq^ method.

ALP Activity Assay

ALP activity was determined both qualitatively and quantitatively. Subconfluent imBMSCs were infected with adenovirus for 24 h and subsequently seeded in 24-well plates. ALP activity was quantitatively assessed using the Great Escape SEAP bioluminescence assay kit (Takara USA Bio) and qualitatively with histochemical staining assay using a mixture of 0.1 mg mL^−1^ naphthol AS-MX phosphate and 0.6 mg mL−1Fast Blue BB salt at specified time points as described^10^. Each assay condition was performed in triplicate.

Matrix Mineralization Assay

Subconfluent imBMSCs were infected with adenovirus and cultured in 24-well cell culture plates in complete DMEM in the presence of ascorbic acid (50 μg/mL) and β-glycerophosphate (10 mM). 14 days post infection, cells were fixed with 0.05% glutaraldehyde at room temperature for 10 min and washed with double distilled water (pH = 4.2). Cells were incubated with 0.4% Alizarin red S (Sigma-Aldrich) at 37 ℃ for 10 min to stain the calcium deposit of the matrix mineralization, and then extensively washed with double distilled water (pH = 4.2). The stain was dissolved in a solution of 20% methanol and 10% acetic acid in water, and quantified by OD reading at 450nm^7^.

Subcutaneous Ectopic Bone Formation

The care and use of animals were approved by the Institutional Animal Care and Use Committee (IACUC) of The University of Chicago (ACUP #71105). All experimental procedures followed the approved guidelines. Athymic nude mice (Envigo, Indianapolis, IN; 6–8-week-old, both male and female) were used for subcutaneous injection experiments. Subconfluent imBMSCs infected with adenovirus were collected 72 hours after infection and resuspended in PPCNg, followed by subcutaneously injected into the flanks of nude mice (3 mice per group, 5 injections per mouse). The mice were sacrificed 28 days post-injection, and all masses at implantation sites were retrieved for histological analysis.

Histological Staining

Retrieved masses were fixed in 4% paraformaldehyde, decalcified, and paraffin-embedded for sectioning. Serial sections of the embedded specimens were mounted onto slides, deparaffinized, and rehydrated, and subsequently subjected to H&E staining, Masson’s Trichome staining and modified PAS staining as previously described^7^.

Data analysis

All experiments were performed at least three times or repeated in three batches of independent experiments. Data were analyzed using GraphPad Prism 7 and presented as the mean ± standard deviations (SD). Statistical significance was determined by one-way ANOVA or the student's t-test for comparison between groups. A value of *P* < 0.05 was considered statistically significant.

**Supplemental Tables**

**Table S1. Tet2 siRNA Oligos**

| siRNA fragment | Oligos |
| --- | --- |
| simTet2-1 Forward | aaaaaCTTGTTCTTCAGCCTCCTTTAtttttAGAGTGGTCT |
| simTet2-1 Fwd w/ BsaI-BbsI-A | ggtGGTCTCGggcaaaaaaCTTGTTCTTCAGCCTCCTTTA |
| simTet2-2 Rev | aaaaaTACAGATGGATTCAGACTCTGtttttTTCGTCCTTTC |
| simTet2-2 Rev w/ BsaI-B | ggtGGTCTCGcgttAaaaaaTACAGATGGATTCAGACTCTG |
| simTet2-3 Rev | aaaaaTTTGTTTCCCGTTTGCATCCTtttttTTCGTCCTTT |
| simTet2-3 Rev w/ BsaI-BbsI-R | ggtGGTCTCGgccaaaaaaTTTGTTTCCCGTTTGCATCCT |

**Table S2. TqPCR Primers Used in Study**

| Gene | Forward primer | Reverse Primer | Accession No. |
| --- | --- | --- | --- |
| Mouse *Col1a1* | GAGCGGAGAGTACTGGATCG | GCTTCTTTTCCTTGGGGTTC | NM_007742 |
| Mouse *Gapdh* | ATGCCATCACTGCCACCC | GCCAGTGAGCTTCCCGTT | NM_001289726.1 |
| Mouse *Opn* | CTCCTCCCTCCCGGTGAA | GCATTCTGTGGCGCAAGG | NM_001204201.1 |
| Mouse *Osx* | TCTCACCAGGTCCAGGCA | GTGTCCCTTGCAGCCCAT | NM_001348205.1 |
| Mouse *Ocn* | CCTTCATGTCCAAGCAGGA | GGCGGTCTTCAAGCCATAC | NM_007541.3 |
| Mouse *Runx2* | AACAAGACCCTGCCCGTG | GCTCCGGCCCACAAATCT | NM_001145920.3 |

**References Cited in Supplemental Materials**

1 Wu, N. *et al.* Overexpression of Ad5 precursor terminal protein accelerates recombinant adenovirus packaging and amplification in HEK-293 packaging cells. *Gene Ther* **21**, 629-637, doi:10.1038/gt.2014.40 (2014).

2 Wei, Q. *et al.* Engineering the Rapid Adenovirus Production and Amplification (RAPA) Cell Line to Expedite the Generation of Recombinant Adenoviruses. *Cell Physiol Biochem* **41**, 2383-2398, doi:10.1159/000475909 (2017).

3 Zhao, G. *et al.* GAPDH suppresses adenovirus-induced oxidative stress and enables a superfast production of recombinant adenovirus. *Genes & Diseases*, 101344, doi:10.1016/j.gendis.2024.101344 (2024).

4 Hu, X. *et al.* CRISPR/Cas9-mediated reversibly immortalized mouse bone marrow stromal stem cells (BMSCs) retain multipotent features of mesenchymal stem cells (MSCs). *Oncotarget* **8**, 111847-111865, doi:10.18632/oncotarget.22915 (2017).

5 He, F. *et al.* FAMSi: A Synthetic Biology Approach to the Fast Assembly of Multiplex siRNAs for Silencing Gene Expression in Mammalian Cells. *Mol Ther Nucleic Acids* **22**, 885-899, doi:10.1016/j.omtn.2020.10.007 (2020).

6 He, T. C. *et al.* A simplified system for generating recombinant adenoviruses. *Proc Natl Acad Sci U S A* **95**, 2509-2514, doi:10.1073/pnas.95.5.2509 (1998).

7 Zhu, Y. *et al.* An Intervertebral Disc (IVD) Regeneration Model Using Human Nucleus Pulposus Cells (iHNPCs) and Annulus Fibrosus Cells (iHAFCs). *Advanced Healthcare Materials* **14**, 2403742, doi:https://doi.org/10.1002/adhm.202403742 (2025).

8 Gou, Y. *et al.* Adipose-derived mesenchymal stem cells (MSCs) are a superior cell source for bone tissue engineering. *Bioact Mater* **34**, 51-63, doi:10.1016/j.bioactmat.2023.12.003 (2024).

9 Zhao, P. *et al.* Effective Bone Tissue Fabrication Using 3D-Printed Citrate-Based Nanocomposite Scaffolds Laden with BMP9-Stimulated Human Urine Stem Cells. *ACS Appl Mater Interfaces* **17**, 197-210, doi:10.1021/acsami.4c13246 (2025).

10 Wang, J. *et al.* Bone morphogenetic protein-9 effectively induces osteo/odontoblastic differentiation of the reversibly immortalized stem cells of dental apical papilla. *Stem Cells Dev* **23**, 1405-1416, doi:10.1089/scd.2013.0580 (2014).
